# Approaches and styles of mothers in sex education process of children and the related factors

**DOI:** 10.1186/s12912-023-01410-w

**Published:** 2023-08-01

**Authors:** Elnaz Faraji Nesfechi, Moluk Pouralizadeh, Zahra Bostani Khalesi, Saman Maroufizadeh

**Affiliations:** 1 Department of Nursing, School of Nursing and Midwifery, Guilan University of Medical Sciences, Rasht, Iran; 2 Shahid Beheshti School of Nursing and Midwifery, Guilan University of Medical Sciences, Hamidyan Ave, Rasht, Iran; 3 Department of Midwifery, School of Nursing and Midwifery, Guilan University of Medical Sciences, Rasht, Iran; 4 Department of Biostatistics and Epidemiology, School of Health, Guilan University of Medical Sciences, Rasht, Iran

**Keywords:** Sex education, Training, Mothers, Communication, Child

## Abstract

**Introduction:**

Sex education supports the development of suitable sexual behaviors in children. However, due to the limitations of sexual issues in Iran, parents may have different sex education styles. This study aimed to assess the styles of mothers in the sex education process of children and the related factors.

**Methods:**

In a cross-sectional study, from March to May 2021, 306 mothers with a 4–12-year-old child who were referred to the comprehensive health service centers in Rasht city were entered into the study by a convenience sampling method. The tool was a questionnaire of parents' sex education styles. Data analysis was performed with independent t-tests, one-way ANOVA, Pearson's correlation coefficient, and the multiple linear regression model using SPSS software version 16.

**Results:**

The majority of the mothers had a mean age of 36.2 ± 6.4 years. The score of the authoritarian sex education style was significantly higher than the other styles (*P* < 0.001). According to the multivariate analysis, 40.6% of the changes in the strict style (R^2^ = 0.406), 32.7% of the changes in the permissive style (R^2^ = 0.327), and 36.1% of the changes in the authoritative style (R^2^ = 0.361) were explained by the personal characteristics of the participants.

**Conclusion:**

The authoritative style was a dominant sex education style. Identifying the factors associated with types of child sex education styles has an important role in promoting the health of children and the community. It is recommended that nursing policymakers identify related factors of sex education styles in mothers of different cultures, and therefore, implement training programs for empowering mothers.

## Lay summary

A cross-sectional study aimed to determine the sex education styles and the related factors in Iranian mothers. According to the results the authoritative style was a dominant style among Iranian mothers. There was no statistically significant difference between strict and permissive style scores

## Introduction

Sex education includes a variety of physical, psychological, mental, and social aspects. It is educating children based on sexual development and their psychological and physical aspects [1]. The purpose of sex education is to support the development of sexual behaviors, the survival of generations, and achieve peace. Today, the general public widely accepts the importance of sex education for children worldwide. Sex education for children is one of the most sensitive subjects of family development [2]. There are different ideas on whether or not children should receive sex education, which topics such an education would include, and at what age such education should be started. In particular, parental awareness of the role they play in the personal development of their children will have a positive effect on their children’s sexual development. A child who receives sex education in phases in a manner appropriate to his/her age would be expected to be more stable in his/her relationships with the opposite sex in later life [3].

Talking to children about sexuality early on establishes it as a normal topic, and avoids awkward and fraught interventions that inevitably occur too late. Secrecy surrounding sex breeds fear and shame, whereas appropriate openness encourages children to ask questions. It is infinitely preferable to have them ask you for answers than try to figure it out on their own only to stumble upon misinformation [2].

In this regard, what can impact the effectiveness of these training courses is to assess the approaches and the styles that parents use for the sex education of their children. Parental sex education styles properly inform the child and help the parents with different attitudes and behaviors in dealing with the child’s sexual development [3].

Various effective factors in the sex education of children included; cultural beliefs, barriers to communication between children and their parents, inadequate skills, negative attitudes, the stigma. These factors create various children's sex education styles [4].

Iran is an Asian country with a majority Muslim population and different cultures, which have a great impact on the lifestyle and beliefs of the people of this country. According to the studies, Iranian parents have, three types of parenting sex education styles, including; authoritative style (good communication between parents and children by providing sex information), strict style (punishment and restriction of the expression of sexual problems), and permissive style (sexually unruly behavior in the children) [3]. Olubunmi et al. defined parenting style as the psychological construct representing standard strategies that parents use in their child-rearing. They reported that parenting styles are categorized into three major forms; authoritative, authoritarian, and permissive parenting styles [5]. Moussa stated the four parenting education styles that encompass many behaviors of parents including authoritative, authoritarian, permissive, and neglectful [6].

Ashcraft and Murray, in their study, showed that, regardless of having the proper knowledge of the parents, their methods for children’s sex education were not appropriate [7]. Regarding Iranian parents’ experiences and the point views on child sex education, Merghati-Khoei et al. showed that due to parents' beliefs in the innocence of children and their inadequate skills in sex education, they were strict in dealing with their children and therefore did not want to have sex education with their children [8].

To the best of our knowledge, the present study is the first research about styles used by Iranian mothers for their children’s sex education. Due to the religious laws in Iran, sexual issues are often considered taboo, so it can be effective in choosing the parent’s sex education styles. In this regard, examining the selected styles of the parents for the sex education of their children and paying attention to the attitude of Iranian parents is so important because it is related to the physical and mental health of children. Therefore, It has an influential role in promoting public and community health and is one of the critical duties of pediatric nurses in children's developmental topics [9]. This study aimed to investigate the approaches and styles of mothers in the sex education process of children and the related factors.

## Materials and methods

### Research design and setting

This study was a cross-sectional study on mothers with 4–12-year-old children who were referred to the comprehensive health service centers in Rasht city (one of the northern cities in Iran).

### Participants and sampling

The participants were selected using the convenience sampling method and 306 mothers who were referred to these centers for vaccination and health care of their children were recruited to this study. All participants who were willing to participate and could understand and speak Persian were included in this study. Exclusion criteria were lack of complete answers to the research questionnaire and refusal to participate during the study period. The sample size was calculated based on the permissive sex education style mean score in the study of Abdollahzadeh and Khosravi [3] with α = 0.05, d = 1, σ = 7.97. Therefore, 244 samples were determined based on the following formula and by considering a 25% attrition rate, 306 mothers were entered into the study.

$$n=\frac{{Z}_{1-\alpha }^{2}/{2}^{{\sigma }^{2}}}{{d}^{2}}$$

$$\alpha =0.05, {z}_{1-\alpha }/{z}_{0.975}=1.95$$

$$\mathrm{n}=\frac{{\mathrm{z}}_{1-\mathrm{\alpha }/2}^{2}{\upsigma }^{2}}{{\mathrm{d}}^{2}}=\frac{{1.96}^{2}{\left(7.97\right)}^{2}}{{1}^{2}}=\frac{244.02}{1}\approx 244$$

### Data collection

The data were collected from all 16 comprehensive health service centers from March to May 2021, in Rasht city. For data collection, the main researcher (that was a master's student in pediatric nursing) visited comprehensive health service centers daily and with the cooperation of the director of the center identified the eligible mothers in relation to the inclusion criteria. After the introduction of the study aims, informed consent was obtained from participants and they were assured of the confidentiality of their personal data.

The data collection tool was a two-part self-report questionnaire. The first part was 27 variables of personal and familial characteristics of the parents and the child including; the mother’s age, father’s age, child’s age, child's educational level, mother’s educational status, father’s educational status, mother’s job, father’s job, mother's ethnicity, father's ethnicity, marital status, duration of marriage (year), number of children, developmental age of the child, child gender, kindergarten attendance experience, mother's supervision on the child's use of technology and mobile phones, child’s birth rank, stepmother, stepfather, child's exposure to sexual, physical and psychological abuse, type of school, child's educational status, living place, financial status, mothers’ participation in sex education training, source of mother's information. We selected the variables based on the literature review.

The second part was a questionnaire containing 33 questions about parents’ sex education styles in children's sex education that was designed and psychometrically evaluated by Abdollahzadeh and Keykhosravi in Iran [10]. This questionnaire consisted of three dimensions strict sex education style (12 questions), permissive sex education style (11 questions), and authoritative sex education style (10 questions). The questions in this questionnaire were based on a five-part Likert scale, from fully disagree (score 0) to fully agree (score 4).

The survey criteria were based on the mean rating for each style. In this study, we measured the reliability of the instrument in a pilot study using 35 samples. Cronbach's alpha coefficients for authoritarian, authoritative, and permissive sex education styles were 0.79, 0.77, and 0.72, respectively, which indicated an acceptable internal consistency. Also, a test–retest with 30 participants was done for the stability of the questionnaire during two weeks which was confirmed with ICC = 0/89.

### Data analysis

In this study, continuous variables were expressed as mean ± standard deviation (SD) and categorical variables as frequency (percentage). Kolmogorov–Smirnov test was used to evaluate the normality of the distribution of variables (P > 0.05). Independent t-tests, one-way ANOVA, and Pearson’s correlation coefficient were used to assess the relationship between sexual parenting style values and personal characteristics of parents, children, and socio-families. Also, in multivariate analyzes, a multiple linear regression model was used to determine the factors related to the scores of sex education styles. In order to compare the mean scores of sexual education styles, the Greenhouse- Geisser test and for Two by two comparison Bonferroni tests were used. Data analysis was performed with SPSS software version 16. A level of 0.05 was considered significant.

## Results

Data from 306 questionnaires were analyzed. The average maternal age was 6.4 ± 36.2 years. A total of 44.4% of the participants had a university education and were housekeepers (68.6%) (Table 1).

**Table 1:** Personal, family and social characteristics of the participants

Frequency (%)	Variables		Frequency (%)	Variables		Mean ± SD**/**Frequency (%)	Variables	
(2.3) 7	Yes	stepfather	(63.4) 194	Gilak	Father's ethnicity	6.4 ± 36.2	Mother’s age	
(97.7) 299	No		(8.8) 27	Talesh		40.5 ± 6.7	Father’s age	
(1.0) 3	Yes	stepmother	(17.6) 54	Turkish		7.9 ± 2.6	Child’s age	
(99.0) 303	No		(10.1) 31	Other		3.5 ± 1.8	Child's educational level	
(3.6) 11	Yes	Child's exposure to sexual, physical and psychological abuse	(95.8) 293	Married	Marital status	(6.9) 21	Primary School	Mother’s educational status
(86.6) 265	No		(4.2) 13	Divorce **/**death of father		(13.7) 42	Secondary School	
(9.8) 30	I don't know		(26.8) 82	10 >	Duration of marriage (year)	(35.0) 107	High School	
(72.8) 142	Public School	Type of school (195 = n)	(35.3) 108	10_15		(44.4) 136	University	
(27.2) 53	Private School		(23.2) 71	15_20		(9.2) 28	Primary School	Father’s educational status
(10.3) 20	Weak / Medium	Child's educational (195 = n) status	(14.7) 45	20 <		(17.3) 53	Secondary School	
(34.9) 68	Good		(37.3) 114	1	Number of children	(28.4) 87	High School	
(54.9) 107	Excellent		(53.3) 163	2		(45.1) 138	University	
(98.4) 301	City	Living place	(9.5) 29	3 ≤		(68.6) 210	Housekeeper	Mother’s job
(1.6) 5	Village		75(24.5)	4–6 Years	Developmental age of the child	(5.9) 18	Self-employment	
(7.5) 23	Bad	Financial status	231(75.5)	6–12 Years		(9.8) 30	Office Employed	
(52.3) 160	Medium		(50.0) 153	Girl	Child gender	(12.4) 38	Medical field	
(40.2) 123	Good		(50.0) 153	Boy		(3.3) 10	Other	
(13.7) 42	Yes	Mothers’ participation in sex education training	(52.3) 160	Yes	Kindergarten attendance experience	(50.3) 154	Self-employment	Father’s Job
			(47.7) 146	No		(28.4) 87	Office Employed	
(86.3) 264	No					(16.3) 50	Labor	
(58.2) 178	Internet, books, television	Source of mother's information	(95.4) 292	Yes	Mother's Supervision on the child's use of technology and mobile phones	(4.9) 15	Medical field	
			(4.6) 14	No		(66.3) 203	Gilak	Mother's ethnicity
(9.8) 30	Friends		(64.4) 197	First	Child 's birth rank	(8.8) 27	Talesh	
(11.1) 34	Field of Study		(29.7) 91	Second		(17.0) 52	Turkish	
(20.9) 64	None		(5.9) 18	Third or fourth		(7.8) 24	Other	

The mean values forstrict, permissive, and authoritative sex education styles were 14.5 ± 8.7, 13.1 ± 6.3, and 30 ± 5.8, respectively. Scores for the authoritative sex education style were significantly higher than authoritarian style (*P* < 0.001) and permissive style (*P* < 0.001), but there was no statistically significant difference between authoritarian and permissive style scores (Table 2).

**Table 2:** Comparison of the mean scores of converted child sex education styles in the participants

Two-by-two comparisons	Mauchly’s Test of Sphericity		Greenhouse-Geiser test		Mean (SD)	Sex education styles
	_(2)_χ^2^	P value	_(1.43 and 434.6)_F	P value		
Permissive < Authoritative strict < Authoritative	157.0	0.001 >	708.6	0.001 >	(18.2) 30.2	Strict style
					(14.3) 29.8	Permissive style
					(14.4) 74.9	Authoritative style

There was a significant relationship between the sex education styles with the type of school (*P* < 0.05) parent's educational status(*P* < 0.01), marital status (*P* < 0.01), number of children (*P* < 0.01), child's birth rank (*P* < 0.01), financial status (*P* < 0.001), ethnicity (*P* < 0.01), mothers' supervision on the child's technology use (*P* < 0.01), mothers' participation in sex education training (*P* < 0.001), and source of mothers' information (*P* < 0.001) (Table 3).

**Table 3:** Relationship between children's sex education styles and personal, family and social characteristics of the participants

Strict sex education style		Permissive sex education style		Authoritative sex education style		Variables	
Mean (SD)	*P* value	Mean (SD)	*P* value	Mean (SD)	*P* value		
(9.9) 27.5	^⁑^0.001 >	(4.9) 17.7	^⁑^0.001 >	(8.7) 24.0	^⁑^0.001 >	Primary School	Mother's educational status
(10.7) 17.4		(6.6) 14.4		(6.0) 29.0		Secondary School	
(7.6) 14.1		(6.9) 12.8		(5.6) 30.3		High School	
(6.6) 12.0		(5.6) 12.2		(4.6) 30.9		University	
(10.8) 23.3	^⁑^0.001 >	(6.6) 16.7	^⁑^0.001 >	(6.8) 26.2	0.001 >	Primary School	Father's educational status
(9.0) 16.2		(6.5) 13.9		(7.4) 29.4		Secondary School	
(8.8) 14.6		(6.3) 13.6		(5.3) 29.8		High School	
(6.7) 12.0		(5.8) 11.8		(4.7) 31.0		University	
(8.5) 14.3	^‡^0.234	(6.2) 13.0	^‡^0.142	(5.6) 30.1	^‡^0.015	Married	Marital status
(12.2) 18.6		(7.7) 15.6		(7.1) 26.2		Divorce / death of father	
(7.7) 13.3	^‡^0.013	(6.3) 12.6	^‡^0.135	(5.0) 30.8	^‡^0.012	Girl	Child 's gender
(9.6) 15.8		(6.3) 13.6		(6.3) 29.1		Boy	
(7.1) 12.7	^⁑^0.004	(6.2) 13.2	^⁑^0.148	(5.2) 30.2	^⁑^0.019	1	Number of children
(9.1) 15.2		(6.3) 12.7		(5.8) 30.3		2	
(11.1) 18.2		(6.4) 15.1		(7.2) 27.1		3 ≤	
(8.30) 14.2	^⁑^0.014	(6.3) 13.4	^⁑^0.056	(5.4) 30.0	^⁑^0.009	First	Child 's birth rank
(8.7) 14.2		(6.2) 12.0		(6.1) 30.6		Second	
(11.5) 20.3		(6.2) 15.6		(6.8) 26.1		Third or fourth	
(8.9) 15.7	^‡^0.001 >	(6.6) 13.7	^‡^0.196	(6.4) 29.2	^‡^0.042	Public School	Type of school (n = 195)
(7.4) 11.0		(5.8) 12.3		(4.9) 31.0		Private School	
(8.2) 16.4	0.006	(6.4) 14.4	^⁑^0.575	(7.5) 28.6	^⁑^0.009	Weak / Medium	Child's educational status (n = 195)
(10.3) 16.7		(6.7) 13.6		(6.4) 28.1		Good	
(7.3) 12.6		(6.3) 12.9		(5.2) 30.9		Excellent	
(8.8) 14.5	^‡^0.975	(6.3) 13.1	^‡^0.972	(5.7) 30.2	^‡^0.142	City	Living place
(7.1) 14.4		(6.8) 13.2		(6.4) 26.2		Village	
(12.1) 23.7	^⁑^0.001 >	(5.2) 17.4	^⁑^0.002	(8.1) 24.6	^⁑^0.001 >	Bad	Financial status
(8.3) 14.6		(6.6) 12.4		(5.8) 30.3		medium	
(7.5) 12.7		(5.8) 13.2		(4.7) 30.5		Good	
(8.1) 13.5	^⁑^0.011	(6.3) 12.9	^⁑^0.867	(5.4) 30.4	^⁑^0.019	Gilak	Mother's ethnicity
(6.8) 15.2		(7.0) 13.1		(6.8) 29.1		Talesh	
(10.9) 18.0		(6.0) 13.8		(6.2) 27.9		Turkish	
(9.3) 14.5		(6.6) 13.0		(5.9) 31.5		Other	
(7.9) 13.6	^⁑^0.003	(6.4) 13.2	^⁑^0.602	(5.3) 30.4	^⁑^0.036	Gilak	Father's ethnicity
(6.8) 13.1		(5.8) 11.8		(5.4) 30.8		Talesh	
(10.8) 18.4		(6.0) 13.7		(6.7) 27.9		Turkish	
(9.7) 15.0		(6.6) 12.6		(6.6) 30.1		Other	
(8.6) 14.2	^‡^0.006	(6.2) 12.9	^‡^0.011	(5.6) 30.2	^‡^0.002	Yes	Mother’s Supervision on the child’s use of technology and mobile phones
(10.5) 20.7		(7.7) 17.3		(7.1) 25.2		No	
(4.8) 10.3	^‡^0.001 >	(5.6) 12.3	^‡^0.379	(4.0) 32.6	^‡^0.001 >	Yes	Mothers’ participation in sex education training
(9.1) 15.2		(6.4) 13.2		(5.9) 29.5		No	
(9.4) 19.7	^⁑^0.001 >	(6.1) 16.3	^⁑^0.001 >	(6.2) 26.4	^⁑^0.001 >	None	Source of mother’s information
(7.5) 12.9		(6.1) 11.8		(5.4) 30.9		Internet, books, television	
(11.1) 18.6		(6.6) 14.9		(5.4) 29.1		Friends	
(4.2) 9.7		(4.9) 12.1		(4.0) 32.5		Field of Study	

^⁑^one-way ANOVA, ^‡^Independent T-test, ^†^Pearson Correlation Coefficient

There were significant correlations between strict and permissive sex education styles and low parenting educational levels, third and fourth children, low-income family finances, lack of sex education information resources, and absence of mothers' supervision on how children use technology and cell phones (*p* < 0.05). There was a significant relationship between strict sex education style with having a son, attending public school, not attending sex education courses, and the parents' Turkish ethnicity (Turkish-speaking parents) (*p* < 0.05) (Table 3).

In multiple linear regression, according to the multivariate analysis, the coefficient of determination (R^2^) was 0.406, which indicates that 40.6% of the changes in strict style scores were explained by the personal characteristics of the parents, the child, and the socio-familial characteristics (Table 4).

**Table 4:** The results of multiple linear regression analysis for factors related to Strict Sex Education Style (*n* = 306)

**b**	**SE**	**β**	**T**	P*	Variables		**b**	**SE**	**β**	**T**	P*	Variables		**b**	**SE**	**β**	**T**	P*	Variables	
-2.40	3.52	-0.04	-0.68	0.496	Yes	Stepfather					Ref	Gilak	Father's ethnicity	0.02	0.24	0.01	0.10	0.923	Child’s age	
														0.32	0.32	0.24	1.00	0.318	Mother’s age	
				Ref	No		-2.34	2.26	-0.08	1.03	0.302	Talesh		-0.54	0.31	-0.42	-1.76	0.080	Father’s age	
-10.99	5.17	-0.12	-2.13	0.034	Yes	Stepmother	0.11	1.96	0.01	0.05	0.957	Turkish		0.64	0.32	-0.23	-1.41	0.159	Mother’s marriage age	
				Ref	No		-1	1.85	-0.03	-0.54	0.590	Other		0.49	0.31	0.25	1.60	0.110	Father’s marriage age	
-1.68	2.45	-0.04	-0.69	0.493	Yes	Child's exposure to sexual, physical and psychological abuse					Ref	Married	Marital status					Ref	Primary School	Mother’s educational status
				Ref	No		4.00	2.49	0.09	1.60	0.110	Divorce **/**death of father		-8.18	2.34	-0.32	-3.50	0.001	Secondary School	
3.69	1.55	0.13	2.38	0.018	I don't know						Ref	10 >	Duration of marriage (year)	-10.36	2.28	-0.57	-4.55	< 0.001	High School	
-4.19	1.25	-0.24	-3.35	0.001	Internet, books, television	Source of mother's information	-0.49	1.38	-0.03	-0.36	0.720	10_15		-9.82	2.55	-0.56	-3.85	< 0.001	University	
							2.09	1.97	0.10	1.06	0.289	15_20						Ref	Primary School	Father’s educational status
							0.72	2.76	0.03	0.26	0.793	20 <		-3.03	1.85	-0.13	-1.64	0.103	Secondary School	
0.63	1.79	0.02	0.35	0.728	Friends															
-5.18	2.10	-0.18	-2.44	0.016	Field of Study						Ref	1	Number of children	-2.11	1.96	-0.11	-1.08	0.282	High School	
				Ref	None		2.67	1.19	0.15	2.25	0.26	2		-3.02	2.20	-0.17	-1.37	0.171	University	
				Ref	City	Living place	1.90	2.65	0.06	0.72	0.474	3 ≤						Ref	Housekeeper	Mother’s job
0.96	3.56	0.01	0.27	0.787	Village		**-2.74**	1.39	-0.11	-1.97	0.050	Yes	Mothers’ participation in sex education training	-2.59	1.98	-0.07	-1.31	0.191	Self-employment	
				Ref	Bad	Financial status					Ref	No		1.67	1.82	0.06	0.92	0.359	Office Employed	
-3.05	1.95	-0.17	-1.56	0.120	Medium						Ref	Girl	Child gender	-1.35	1.94	-0.05	-0.70	0.486	Medical field	
-2.93	2.11	-0.16	-1.39	0.166	Good		1.95	0.89	0.11	2.19	0.03	Boy		2.422	2.60	0.05	0.93	0.353	Other	
							-0.12	0.97	-0.01	-0.13	0.901	Yes	Kindergarten attendance experience					Ref	Self-employment	Father’s Job
											Ref	No		-0.17	1.23	-0.01	-0.14	0.893	Office Employed	
														-0.53	1.37	-0.02	-0.39	0.698	Worker	
							-3.18	2.24	-0.08	-1.42	0.156	Yes	Mother's Supervision on the child's use of technology and mobile phones	-0.39	2.32	-0.01	-0.17	0.866	Medical field	
											Ref	No						Ref	Gilak	Mother's ethnicity
											Ref	First	Child’s birth rank	3.16	2.19	0.10	1.44	0.150	Talesh	
							-1.59	1.40	-0.08	-1.13	0.258	Second		-1.02	1.96	-0.04	-0.52	0.602	Turkish	
							-0.35	3.47	-0.01	-0.10	0.920	Third or fourth		2.85	2.05	0.09	1.39	0.167	Other	

*B* Regression coefficient, *SE* Standard Error; R^2^: 0.406; * Linear regression analysis

In relation to the permissive sex education style, the coefficient of determination (R^2^) was 0.327, which indicates 32.7% of the changes in this style were explained by the personal characteristics of the participants (Table 5). In the authoritative sex education style, R^2^ = 0.361 showed that 36.1 of the changes were explained by the personal characteristics of the study participants (Table 6).

**Table 5:** The results of multiple linear regression analysis for factors related to permissive Sex Education Style

**b**	**SE**	**β**	**T**	P*	Variables	
0.14	0.18	0.06	0.76	0.451	Child’s age	
0.12	0.25	0.12	0.47	0.639	Mother’s age	
-0.20	0.24	-0.21	-0.82	0.410	Father’s age	
0.01	0.25	0.01	0.02	0.986	Mother’s marriage age	
0.17	0.24	0.12	0.71	0.481	Father’s marriage age	
				Ref	Primary School	Mother’s educational status
-3.01	1.79	-0.16	-1.68	0.094	Secondary School	
-2.95	1.75	-0.22	-1.69	0.92	High School	
-2.42	1.96	-0.19	-1.24	0.217	University	
				Ref	Primary School	Father’s educational status
1.03	1.42	-0.06	-0.73	0.468	Secondary School	
-0.82	1.51	-0.06	-0.54	0.586	High School	
-2.76	1.69	-0.22	1.63	0.103	University	
				Ref	Housekeeper	Mother’s job
-2.77	1.52	-0.10	-1.82	0.069	Self-employment	
-2.30	1.40	-0.11	-1.64	0.102	Office Employed	
-2.06	1.49	-0.11	-1.38	0.168	Medical field	
1.26	2.00	0.04	0.63	0.528	Other	
				Ref	Self-employment	Father’s Job
-0.22	0.94	-0.02	-0.24	0.813	Office Employed	
-2.17	1.05	-0.13	-2.06	0.040	Worker	
0.24	1.78	0.01	0.14	0.892	Medical field	
				Ref	1	Number of children
-0.43	0.91	-0.03	-0.47	0.640	2	
0.48	2.04	0.02	0.23	0.815	3 ≤	
				Ref	Married	Marital status
1.46	1.91	0.05	1.76	0.447	Divorce **/**death of father	
				Ref	10 >	Duration of marriage (year)
-0.60	1.06	-0.05	-0.57	0.571	10_15	
-0.74	1.51	-0.05	-0.49	0.625	15_20	
1.31	2.12	0.07	0.62	0.537	20 <	
				Ref	Girl	Child gender
1.45	0.68	0.12	2.13	0.035	Boy	

*B* Regression coefficient, *SE* Standard Error; R^2^.: 0.327; * Linear regression analysis

**Table 6:** The results of multiple linear regression analysis for factors related to authoritative Sex Education Style (*n* = 306)

**b**	**SE**	**β**	**T**	P*	Variables		**b**	**SE**	**β**	**T**	P*	Variables		**b**	**SE**	**β**	**T**	P*	Variables	
1.65	2.41	0.04	0.69	0.493	Yes	Stepfather					Ref	Gilak	Father's ethnicity	-0.12	0.16	-0.05	-0.72	0.471	Child’s age	
														0.25	0.22	0.28	1.15	0.253	Mother’s age	
				Ref	No		1.94	1.55	0.10	1.26	0.211	Talesh		-0.07	0.21	-0.09	-0.35	0.725	Father’s age	
3.64	3.53	0.06	1.03	0.304	Yes	Stepmother	-0.88	1.34	-0.06	-0.66	0.512	Turkish		-0.30	0.22	-0.23	-1.34	1.82	Mother’s marriage age	
				Ref	No		0.84	1.26	0.04	0.66	0.508	Other		0.08	0.21	0.06	0.38	0.705	Father’s marriage age	
-2.51	1.68	-0.08	-1.50	0.135	Yes	Child's exposure to sexual, physical and psychological abuse					Ref	Married	Marital status					Ref	Primary School	Mother’s educational status
				Ref	No		-2.96	1.71	-0.10	-1.73	0.084	Divorce **/**death of father		3.74	1.60	0.22	2.34	0.020	Secondary School	
-3.24	1.06	-0.17	-3.06	0.002	I don't know						Ref	10 >	Duration of marriage (year)	3.79	1.56	0.31	2.44	0.016	High School	
3.67	0.85	0.31	4.29	< 0.001	Internet, books, television	Source of mother's information	-0.62	0.94	-0.05	-0.66	0.508	10_15		2.70	1.74	0.23	1.55	0.123	University	
							-2.66	1.34	-0.19	-1.98	0.049	15_20						Ref	Primary School	Father’s educational status
							-4.13	1.89	-0.25	-2.19	0.030	20 <		0.60	1.27	0.04	0.48	0.634	Secondary School	
2.91	1.23	0.15	2.38	0.018	Friends															
5.30	1.44	0.29	3.69	< 0.001	Field of Study						Ref	1	Number of children	-0.14	1.34	-0.01	-0.10	0.918	High School	
				Ref	None		0.84	0.81	0.07	1.03	0.304	2		0.29	1.50	0.030	0.19	0.846	University	
				Ref	Bad	Financial status	-0.12	1.81	-0.01	-0.06	0.949	3 ≤						Ref	Housekeeper	Mother’s job
3.28	1.34	0.28	2.46	0.015	Medium		2.13	0.95	0.13	2.24	0.026	Yes	Mothers’ participation in sex education training	2.59	1.35	0.11	1.92	0.056	Self-employment	
2.88	1.44	0.25	1.99	0.047	Good						Ref	No		1.46	1.25	0.08	1.17	0.241	Office Employed	
											Ref	Girl	Child gender	0.95	1.32	0.05	0.72	0.473	Medical field	
							-1.26	0.61	-0.11	-2.07	0.04	Boy		-0.41	1.78	-0.01	-0.23	0.816	Other	
											Ref	City	Living place					Ref	Self-employment	Father’s Job
							**-4.32**	2.44	-0.10	-1.77	0.078	Village		-0.32	0.84	-0.03	-0.38	0.702	Office Employed	
														1.15	0.94	0.07	1.23	0.219	Worker	
							2.38	1.53	0.09	1.56	0.121	Yes	Mother's Supervision on the child's use of technology and mobile phones	0.22	1.59	0.01	0.14	0.890	Medical field	
											Ref	No						Ref	Gilak	Mother's ethnicity
											Ref	First	Child 's birth rank	-2.91	1.50	-0.14	-1.94	0.053	Talesh	
							0.80	0.96	0.06	0.84	0.404	Second		-0.02	1.34	-0.01	-0.02	0.988	Turkish	
							-0.60	2.37	-0.02	-0.25	0.802	Third or fourth		0.53	1.40	0.02	0.37	0.709	Other	

B: Regression coefficient; SE: Standard Error; R^2^:0.361; * Linear regression

## Discussion

In the current study, the majority of Iranian mothers followed the authoritative sex education style. This result is consistent with the study of Shin et al. [2] and Binti Abdullah et al. [10]. While it is inconsistent with the study of Nasution et al., that reported mothers with strict views considered sex education taboo and believed that society considered issues related to sex education as abnormal and, therefore, their embarrassment senses prevented them from adequately teaching their children [11]. In the other study by Merghati-Khoei et al. In Iran, parents believed that children could be protected by strict sex education, which is inconsistent with the results of the current study [12]. Therefore, in a strict sex education style, parents have a closed view of the sex education of their children. Such cases may be associated with negative consequences such as future behavioral and moral deviations of the children [3]. These differences may be due to culture and beliefs that are different even between people of one nation.

The results showed in the parents with higher education levels, the score of authoritative sex education style increased significantly, but the scores of strict and permissive styles decreased significantly. The higher educated mothers that had an authoritative style also reported that their source of information about sex education was their field of study, but the participants in both strict and permissive sex education styles did not use any information source. Along with the present study, we can mention the studies of Vaghari et al. [13] and Faizah et al. [14]. Advanced personal knowledge and general information can affect personal thinking, attitudes, and perceptions [13].

In a study by Martin et al., Higher educated mothers showed a better attitude toward their children's sex education, while less-educated mothers showed a more closed or even more permissive attitude towards their children's sex education [15].

According to the results, mothers who followed the authoritative style of sex education had a higher socioeconomic level than the two strict and permissive styles, which means that they were in a better financial condition and their child was in a lower birth rank. The majority of these mothers lived with their spouses and had better control over their children's technology and mobile phone use. The study findings of Asuquo et al. [16] in Nigeria is consistent with the findings of the present study.

In contrast to the current study, Devi and Yadav showed no significant correlation between the sex education style of rural parents and the family's financial status [17].

This difference seems to be related to cultural differences between urban and rural communities. Work diversity may be less in rural areas than in urban areas. In general, rural parents have a stronger taboo on sex education and related topics and may have much less access to public resources on sex education [18].

This study indicated that mothers who followed an authoritative sex education style had more control over their children's technology and mobile phone use. A study by Keikha et al. [19] is consistent with this finding. In the study of Ihmeideh and Shawareb, parents who had a strict style set hard rules for their children to use the Internet. They punished their children for connecting to social media [20].

In this study, mothers with an authoritative style had fewer children than mothers with a strict style. In the majority of these mothers, the children under study were daughters who were under education in private (non-public) schools. They were the first child of their family. Also, most of these mothers had participated in sex education training.

A study by Zedan et al. showed that the more children a family has, the less care, support, and encouragement they have [21] which is in line with the present study. One of the inconsistent results is the study of Sourinejad et al. that indicated mothers with two or more children have higher authoritative style scores than single mothers [22]. Attitudes and expectations of parents change through the experience of the first child.

Parental behavior with the first child seems different from the next child. Of course, if the family's financial status is complicated, parents may have limited time to meet the educational needs of all their children, and many school-aged children may start working to support the family's financial situation. This issue reduces family oversight and support for children [23].

According to the results of this study, most mothers that had one son followed a strict style of sex education, while mothers with one daughter followed an authoritative style.

Regarding the authoritative style of sex education in the participants and the gender of their children, Purwanti et al. showed that parents have a deeper relationship with their daughters than their sons when they educate their children, which is consistent with current studies [24]. However, Sharifi et al. reported that a strict sex education style is more common among parents with daughters than mothers with sons [25] which is inconsistent with the current study. This indicates that Iranian families with girl children have more concern about the sex education of their children.

Comparing two strict and authoritative styles showed that the majority of children that had mothers with authoritative and strict styles were educated in private schools (non-public) and public schools respectively. In this regard, a study by Qarebaghi et al. found that most mothers with children in public schools adhered to a strict sex educational style [26]. In contrast to the current study, McKay et al. showed that there was not much difference in the type of answers given about sex education between students' parents in public and private (non-public) schools. They showed that parents whose children attending in Catholic schools have shown a stricter attitude towards sex education, but actually, there was no difference between the parents of public and Catholic schools [27].

Comparing the two strict and the authoritative styles, we found that mothers with the authoritative styles had more participation in the sex education classes and had more information than the others. Mothers who followed a strict or permissive sex education style had less information on issues related to their child's exposure to sexual abuse and harassment. Consistent with the current study in the study by Lo et al. the parents who had authoritative educational views were more aware of the sex education of children and various types of child abuse than other views [28]. In their study, Khanjari et al. found that 20% of mothers had a permissive sex education style [29].

According to the results, a significant association was observed between authoritative style and marital status and most authoritative mothers lived with their husbands. Regarding the study of Jamaluddin which was along with the present study, the divorced parents had a lower level of knowledge about sex education of their children than the married parents [24].

Rosenkrantz and Houston concluded that mothers living alone for some reason are more violent and somewhat stricter than their children [30].

This study has some limitations including the cross-sectional design and self-report responses.

Since this study was conducted during the pandemic of COVID-19, various factors, including social distance, may have reduced the tendency of mothers to stay longer in comprehensive health service centers, affecting the accuracy of answering questions. The results of this study and the participants’ attitudes may also be influenced by Iran's Islamic culture and the related teachings about the limitations of relationships between the opposite genders.

## Conclusion

According to the results, the authoritative style was reported as a dominant sex education style in parents who participated in this study. Also, the child's gender, type of school, parent's educational status, marital status, number of children, child's birth rank, parent's educational status, financial status, ethnicity, parent’s supervision on the child's technology use, parent’s participation in sex education training, and Source of parent's information were the related factors with the type of the sex education style. Since parents' sex education styles are the most important factors in shaping children's personalities, ensuring family health, and promoting community health, therefore, we recommend nursing managers and policymakers to identify other types of child sex education styles and the factors associated with different communities because it can change greatly under the influence of the different cultures. Since the role of pediatrics’ nurses is to promote health in all three levels of prevention, they are the most suitable people to evaluate and identify this important issue at the community level. This will be made possible by promoting evidence-based practices in sex education and repeating the research in different communities. Identifying the inappropriate and harmful styles of sexual education of children by their mothers, which often occurs due to their lack of awareness, health policymakers can prevent the consequences of this by supporting the implementation of specialized educational and counseling interventions for parents. Ensuring healthy sexual development in children can have an impact on the health of society.

## Data Availability

The data that support the findings of this study are available from the corresponding author upon reasonable request.
